# Recombinant α-NAcetylgalactosaminidase from Marine Bacterium-Modifying A Erythrocyte Antigens

**Published:** 2015

**Authors:** L. A. Balabanova, V. A. Golotin, I. Y. Bakunina, L. V. Slepchenko, V. V. Isakov, A. B. Podvolotskaya, V. A. Rasskazov

**Affiliations:** G.B. Elyakov Pacific Institute of Bioorganic Chemistry, Far Eastern Branch, Russian Academy of Sciences, 100-letiya Vladivostoka Ave., 159, 690022, Vladivostok, Russia; Far Eastern Federal University, Sukhanova Str., 8, 690950, Vladivostok, Russia

**Keywords:** glycoside hydrolase GH109, Arenibacter latericius, 1H NMR spectroscopy, conversion of A erythrocytes

## Abstract

A plasmid based on pET-40b was constructed to synthesize recombinant α-N-acetylgalactosaminidase of the marine bacterium *Arenibacter latericius *KMM 426T (α-AlNaGal) in *Escherichia coli *cells. The yield of α-Al- NaGal attains 10 mg/ml with activity of 49.7 ± 1.3 U at 16°C, concentration of inductor 2 mM, and cultivation for 12 h. Techniques such as anion exchange, metal affinity and gel filtration chromatography to purify α-*Al*NaGal were applied. α-*Al*NaGal is a homodimer with a molecular weight of 164 kDa. This enzyme is stable at up to 50°C with a temperature range optimum activity of 20–37°C. Furthermore, its activity is independent of the presence of metal ions in the incubation medium. 1H NMR spectroscopy revealed that α-*Al*NaGal catalyzes the hydrolysis of the O-glycosidic bond with retention of anomeric stereochemistry and possesses a mechanism of action identical to that of other glycoside hydrolases of the 109 family. α-*Al*NaGal reduces the serological activity of A erythrocytes at pH 7.3. This property of α-*Al*NaGal can potentially be used for enzymatic conversion of A and AB erythrocytes to blood group O erythrocytes.

## INTRODUCTION

α-N-Acetylgalactosaminidases (EC 3.2.1.49) catalyze the removal of 2-acetamido-2-deoxy-*D*-glucopyranosyl residues bound via the α-O-glycosidic bond (Gal- NAcα) from the non-reducing ends of oligosaccharides and glycoconjugates: in particular agglutinogens of the human blood groups A and AB. α-N-Acetylgalactosaminidases can be used to study the structure of natural compounds and synthesize new oligosaccharides [1]. The study of α-N-acetylgalactosaminidases is also associated with their involvement in the catabolism of complex oligosaccharides in the human body [2]. The practical interest in the enzyme has stemmed from the fact that it can potentially be used for enzymatic conversion of the blood groups A and AB to the universal blood group O via deglycosylation of antigenic determinants [3]. For this purpose, glycoside hydrolases of family 27 (GH27) from chicken liver and family 36 (GH36) from *Clostridium perfringens *bacterium were isolated [4, 5]. These enzymes have a number of disadvantages for biotechnological application, such as an unphysiological pH optimum and inefficiency in converting erythrocytes of subtype A_1_.

α-N-Acetylgalactosaminidase of *Arenibacter latericius* KMM 426^T^, which effectively inactivates the serological activity of the A1 and A2 antigens of erythrocytes at neutral pH, was discovered by screening 3,000 strains of marine bacteria [6, 7]. Based on the classification of structural homology, α-N-acetylgalactosaminidase of *Arenibacter latericius *KMM 426T is classified as belonging to the glycoside hydrolase family 109 (GH109) [8, 9].

A method for synthesizing recombinant α-N-acetylgalactosaminidase (α‑*Al*NaGal) to study its enzymatic properties is suggested in this work.

The nucleotide sequence of the α-*Al*NaGal gene was amplified from the genomic DNA of marine bacterium* A. latericius *type strain KMM 426^T^ using primers: Nac40_NcoF (5'-TTAACCATGGAAAATCTTTATTTTCAGGGTGGGGCTAAGTACATGGGCGGTTTTTCTGCT- 3') and Nac40_SalIR (5'-TTAAGTCGACACCCTGAAAATAAAGATTTTCGCTTA CAATATCTAATGGTGCAGTGGT-3') (Eurogene). PCR amplification was performed in an Eppendorf amplifier using the following program: 95°C for 2 min and 35 cycles of 95°C for 15 s, 72°C for 1 min. The α‑*Al*NaGal gene was cloned into vector pET-40b(+) (Novagen) at the NcoI–SalI restriction sites after the DsbC sequence and His-tag. Recombinant plasmids were obtained in* Escherichia coli *DH5α cells. The α‑*Al*NaGal-producing strain was obtained by transformation of plasmid into* E. coli *Rosetta(DE3). An overnight culture of the producing strain was grown in a 1-l flask with a liquid LB medium (pH 7.7) containing 25 mg/ml of kanamycin at 37°C and shaking at 200 rpm. When the culture reached the OD_600_ of 0.6–0.8, it was induced with 0.2 mM isopropyl- β-D-1-thiogalactopyranoside (IPTG) and incubated at 16°C for 12 h.

Activity of α‑*Al*NaGal was determined according to the cleavage of *p*-nitrophenyl-α-N-acetylgalactosaminide. The reaction mixture (400 μl) contained 10 mM NaH_2_PO_4_, pH 7.2, 3 mM substrate, and the enzyme. After 20 min of incubation at 20°C, the reaction was terminated by adding 0.6 ml of 1-M Na_2_CO_3_. Absorbance at 400 nm was used to calculate the amount of the released product. One unit of activity (U) was defined as the amount of enzyme catalyzing the formation of 1 μM of *p*-nitrophenol per minute. Specific activity was estimated as units of enzyme activity per milligram of the protein. Protein concentration was determined according to the Bradford method. The yield of the total enzyme activity was 49.7 ± 1.3 U per 1 l of culture broth.

**Fig. 1:**
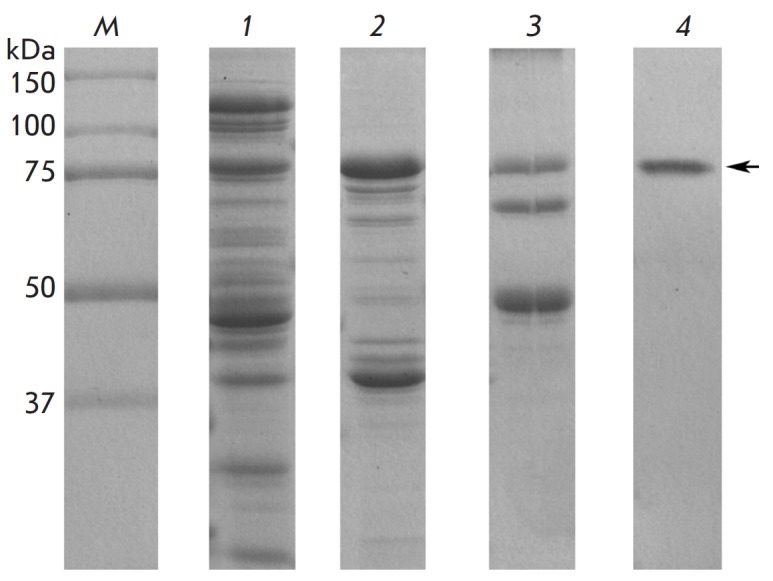
The expression and purification of α-*Al*NaGal (12% SDS-PAGE): **M **– protein molecular weight marker (Bio-Rad); **1 **–whole-cell extract; **2 **–DEAE-MacroPrep;** 3 **– Ni-agarose; **4 **– Sephacryl S-200HR. Migration of α-*Al-* NaGal is marked with an arrow

**Fig. 2:**
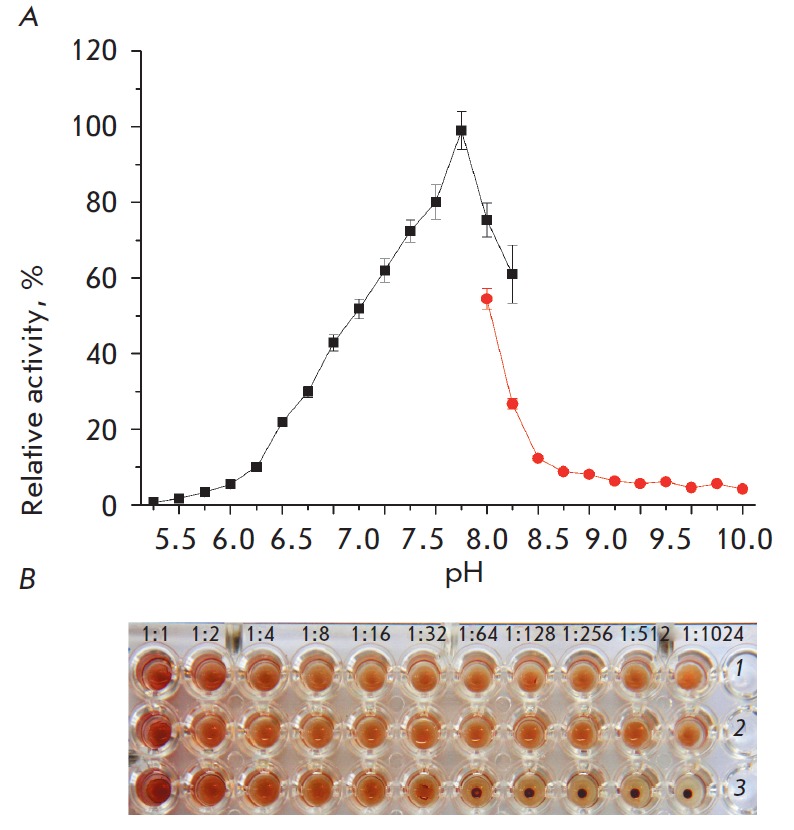
Enzymatic properties of α-*Al*NaGal: **A **– optimum pH of α-*Al*NaGal; **B **–1% suspension of A erythrocytes mixed with anti-A serum in a series of double-dilution steps in: **1 **–20 mM Na+-phosphate buffer, **2 **–20 mM glycine- NaOH buffer, **3 **–20 mM Na+-phosphate buffer after treatment with α-*Al*NaGal

Purification of α‑*Al*NaGal was carried out at +6°C.* E. coli *cells were centrifuged at 5,000 rpm for 10 min, re-suspended in 200 μl of buffer A (0.01 M NaH_2_PO_4_, pH 7.8, 0.01% NaN_3_), and sonicated using a UZDN 2-T ultrasonic disperser (USSR). The solution was centrifuged (25 min, 11,000 rpm) and added to the column (2.5 × 37 cm) containing a DEAE-MacroPrep ion exchange resin (Bio-Rad) equilibrated with buffer A. Elution was performed with a linear gradient of 0–0.25 M NaCl in buffer A. The active fractions were collected and loaded onto a column (1 × 2 cm) with Ni-agarose (Qiagen). The protein was eluted using 50 mM EDTA. The eluate was loaded onto a Sephacryl S-200HR (Sigma) gel filtration column equilibrated with buffer A. Homogeneity of α‑*Al*NaGal was confirmed using a 12% polyacrylamide gel in the presence of sodium dodecyl sulfate (SDS-PAGE) (*Fig. 1*). The results of gel filtration revealed that α‑*Al*NaGal is a homodimer with a molecular weight of 164 kDa (96 kDa after DsbC plasmid sequence at the site of enterokinase (Novagen) had been removed). The enzyme is stable at up to 50°C with a temperature range of optimum activity of 20–37°C, while its activity is independent of the presence of metal ions in the incubation medium. The additional amino acid residues have no influence on the enzymatic properties; therefore, their removal can be neglected. The optimum pH was determined in 20 mM Na^+^-phosphate and glycine-NaOH buffers at intervals of pH 5.4–8.2 and 8.0–10.0 (*Fig. 2A*). The study of the α‑*Al*NaGal properties revealed a possibility of usage to deglycosylate blood group A erythrocyte determinants (blood transfusion station, Vladivostok) at neutral pH. Blood group A erythrocytes were washed with a normal saline solution and then diluted with a Na+-phosphate isotonic buffer to a final concentration of 20%. 0.02 ml of the obtained suspension was mixed with 0.08 ml of the α‑*Al*NaGal solution (0.004 U) in the same buffer. After 24 h of incubation at 26°C, erythrocytes were washed three times using the same buffer (pH 7.3) with gentle shaking. A 1% suspension was prepared and then mixed with an anti-A serum (Mediclon, Russia) in a series of double-dilution steps in 96- well plates (Costar). After 1 h of incubation at room temperature, agglutination titer was measured (*Fig. 2B*). The results of an immunological analysis showed that the serological activity of A antigens of erythrocytes treated with α‑*Al*NaGal decreases as a result of their enzymatic transformation to H antigens, because no agglutination was observed up to a titer of 1/16. α‑*Al*NaGal causes neither nonspecific aggregation of erythrocytes nor their hemolysis.

The enzyme of marine bacterium *Arenibacter latericius* type strain KMM 426^T^ can fully inactivate the serological activity of A erythrocytes at neutral pH and compares favorably with α-N‑acetylgalactosaminidases from chicken liver and *C. perfringens*, which affect only the A2 subgroup of erythrocytes [5, 6]. Being classical hydrolases, the GH27 and GH36 enzymes catalyze the hydrolysis of the O-glycosidic bond of their substrate via the double displacement mechanism with retention of the stereochemistry of the anomeric center of the substrate [10]. More recently, an enzyme of a new GH109 family has been isolated from pathogenic bacterium* Elizabethkingia meningoseptica*. This enzyme had properties similar to those of α-N‑acetylgalactosaminidase of the *A. latericius *type strain KMM 426^T^ and a different mechanism of hydrolysis of the classical hydrolases [8]. The mechanism includes stages of elimination of the O-glycosidic bond and proton exchange at C_2_ of N-acetylgalactosamine with retention of anomeric stereochemistry.

**Fig. 3:**
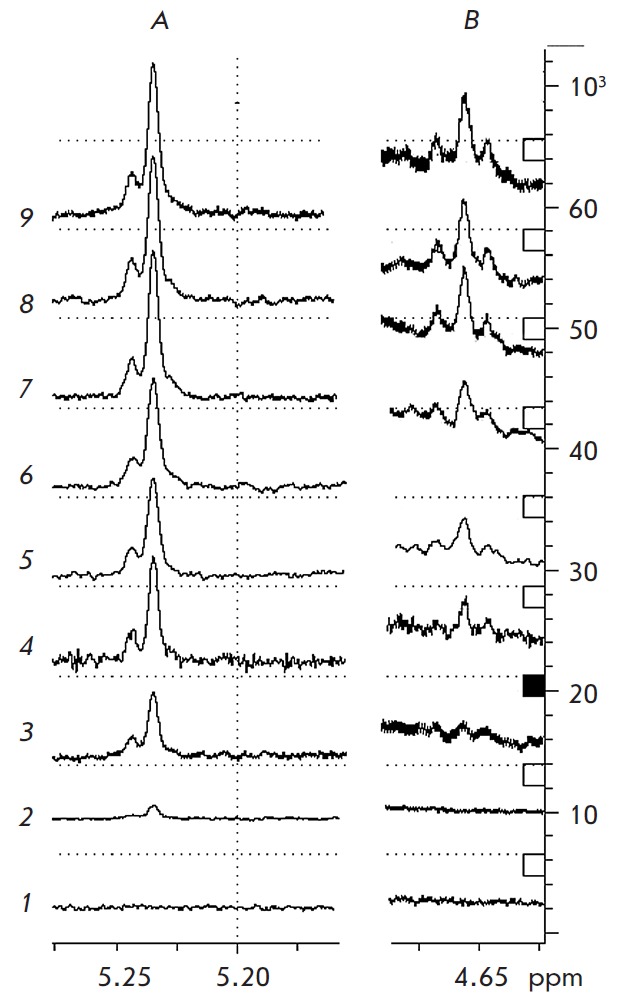
The resonance regions Δδ=5.30–5.20 ppm (**A**) and Δδ=4.75–4.10 ppm (**B**) of ^1^H NMR spectrum of α- and β-anomeric atoms of N-acetylgalactosamine as a product of α-*Al*NaGa hydrolysis for 0 min (1), 10 min (2), 20 min (3), 30 min (4), 40 min (5), 50 min (6), 80 min (7), 90 min (8), 100 min (9)

The configuration of the anomeric center of the hydrolysis products of α‑*Al*NaGal was directly examined using ^1^H NMR spectroscopy. The experiment was carried out at 20°C using a DRX-500 NMR spectrometer (Bruker). ^1^H NMR spectra were acquired using a spectral width of 5,000 Hz over 32,000 data points. Prior to the analysis, 0.6 ml of a 50 mM Na^+^-phosphate solution (pH 7.5) containing 6.0 mM *p*-nitrophenyl-α-N-acetylgalactosaminide substrate was evaporated and dissolved in 0.6 ml of D_2_O. The deuterium-exchanged α‑*Al*NaGal was obtained using Vivaspin turbo 10 k MWCO columns (Sartorius). Chemical shifts in spectra were referenced to acetone (δ = 2.22 ppm) in D_2_O used as an external standard. After measuring the initial spectra of the substrate at t = 0 min, 0.1 ml of the deuterium-exchanged α‑*Al*NaGal, containing 0.98 U, was added to 6.0 mM of the deuterium-exchanged* p*-nitrophenyl-α-N-acetylgalactosaminide in 0.6 ml D_2_O to initiate the reaction. The ^1^H NMR spectra were automatically recorded at 10 min intervals for 180 min after the onset of the reaction. *Fig. 3* shows the resonance regions Δδ=5.30–5.20 ppm and Δδ=4.75–4.10 ppm of the ^1^H NMR spectrum of the reaction products. The product, with a resonance signal at 5.22 ppm, is formed during the first minutes after enzyme addition (*Fig. 3A*). This signal corresponds to the proton of the anomeric center of unbound N‑acetylgalactosamine (GalNAcα). Signal intensity increases during the following 10 min of the reaction. The signal of the β-anomer of GalNAcα with the chemical shift at 4.64 ppm as a result of mutarotation appears only after 20 min of the reaction’s onset (*Fig. 3B*). The spectra of α- and β-anomers of unbound GalNAcα show that the signals are observed as doublets with SSCC of 3.8 and 7.8 Hz, and a singlet. These observations indicate that proton– deuterium substitution takes place at C2. Such a catalytic mechanism is typical of glycoside hydrolases GH109 [8, 11].

## CONCLUSIONS

The recombinant protein α-*Al*NaGal with a molecular weight of 164 kDa, with the properties of α-N-acetylgalactosaminidase of marine bacterium *A. latericius* type strain KMM 426T, was synthesized. α-*Al*NaGal catalyzes the hydrolysis of the α-O-glycosidic bond with retention of the stereochemistry of the anomeric center of the substrate and proton exchange to deuterium of the solvent at C2 via a mechanism typical of glycoside hydrolases of the GH109 family. α-*Al*NaGal deglycosylates A antigens of the blood at pH 7.5. This property demonstrates that α-*Al*NaGal can be used to obtain blood group O erythrocytes.
